# Repression of AKT signaling by ARQ 092 in cells and tissues from patients with Proteus syndrome

**DOI:** 10.1038/srep17162

**Published:** 2015-12-11

**Authors:** Marjorie J. Lindhurst, Miranda R. Yourick, Yi Yu, Ronald E. Savage, Dora Ferrari, Leslie G. Biesecker

**Affiliations:** 1 National Human Genome Research Institute, National Institutes of Health, Bethesda, MD, USA; 2 ArQule, Inc., Burlington, MA, USA

## Abstract

A somatic activating mutation in *AKT1,* c.49G>A, pGlu17Lys, that results in elevated AKT signaling in mutation-positive cells, is responsible for the mosaic overgrowth condition, Proteus syndrome. ARQ 092 is an allosteric pan-AKT inhibitor under development for treatment in cancer. We tested the efficacy of this drug for suppressing AKT signaling in cells and tissues from patients with Proteus syndrome. ARQ 092 reduced phosphorylation of AKT and downstream targets of AKT in a concentration-dependent manner in as little as two hours. While AKT signaling was suppressed with ARQ 092 treatment, cells retained their ability to respond to growth factor stimulation by increasing pAKT levels proportionally to untreated cells. At concentrations sufficient to decrease AKT signaling, little reduction in cell viability was seen. These results indicate that ARQ 092 can suppress AKT signaling and warrants further development as a therapeutic option for patients with Proteus syndrome.

Proteus syndrome is characterized by progressive, mosaic, segmental overgrowth that can affect any organ or tissue in the body^1^. It is caused when a c.49G>A, p.Glu17Lys (hereafter referred to as AKT1 E17K) somatic activating mutation^2^ in the serine/threonine kinase *AKT1* occurs during development and results in an individual with both mutant and wild type cells^3^. The overgrowth observed in individuals with Proteus syndrome is typically asymmetric, begins postnatally, progresses rapidly and disproportionately, and often results in distortion of the normal tissue. The severity and extent of tissue overgrowth varies greatly, with each patient manifesting a unique combination of abnormalities. Tissues such as bone, fat, skin, and connective tissue are more typically involved. Cerebriform connective tissue nevi (CCTN), asymmetric, distorting bony overgrowth, vascular anomalies, and dysregulation of fatty tissue are common manifestations of this condition. Additionally, affected patients have a predisposition to benign and malignant tumors including mesothelioma, breast cancer^4^,^5^,^6^, and papillary thyroid carcinoma (Doucet *et al.*, unpublished observations). However, the most common life threatening manifestations are deep vein thrombosis and pulmonary embolism^7^. Currently, there are no drugs approved for the treatment of patients with Proteus syndrome, and the only known treatments are symptomatic.

The pathophysiology of Proteus syndrome links overgrowth to malignancy through the phosphatidylinositol 3 kinase (PI3K)/AKT signaling pathway^2^,^3^. AKT is activated when it translocates to the cell membrane by the binding of its plekstrin homology (PH) domain to the second messenger phosphatidylinositol 3,4,5-trisphosphate [PI(3,4,5)P_3_]. Once this interaction occurs, AKT is phosphorylated at threonine 308 (T308) by phosphoinositide-dependent kinase 1 (PDK1, encoded by *PDPK1*) which also binds PI(3,4,5)P_3_. AKT becomes fully active when the serine residue at position 473 (S473) is phosphorylated by the mTORC2 complex. In quiescent cells, levels of PI(3,4,5)P_3_ are low and AKT is inactive. Upon growth factor stimulation, PI3K is activated and converts the more abundant phosphatidylinositol 4,5-bisphosphate [PI(4,5)P_2_] to PI(3,4,5)P_3_, which is then available for AKT activation. *In vitro* studies have shown that the presence of the E17K mutation in AKT1 increases its affinity for PI(3,4,5)P_3_ sevenfold and PI(4,5)P_2_ greater than 100-fold over wild-type AKT^8^ and that it also weakens the interaction between the PH and kinase domains of AKT that occurs when AKT is inactive^9^. These studies predict that mutant AKT will remain phosphorylated even in the absence of growth factor signaling. In Proteus syndrome cells this is indeed the case as pAKT levels were markedly higher than in controls when cells were grown in serum-free medium^3^.

AKT is part of the PI3K/AKT signaling pathway that regulates many cellular processes including cell growth, proliferation and apoptosis^10^. As such, mutations in these genes often result in up regulation of this pathway found in many cancerous tumors. However, unlike in cancer cells where dozens of driver and passenger mutations accumulate in many genes that disrupt numerous cellular functions, Proteus cells are thought to contain only the E17K AKT1 mutation, making these cells an attractive system for studying the effects of a single perturbation on cell growth and metabolism. This implies that therapeutic agents for treating patients with Proteus syndrome would only need to reduce the effects of the exaggerated AKT1 signaling, which is in contrast to cytotoxic cancer treatments that are designed to kill the cells. It is likely that individuals with Proteus syndrome will need to continue treatment for many years, necessitating the development of drugs that are well tolerated and easy to administer. Identification of agents that can reduce the effects of constitutive activation of AKT without significant toxicity will be key to developing treatments for Proteus syndrome.

ARQ 092 is a novel, orally bioavailable non-ATP competitive allosteric pan-AKT inhibitor. It is highly selective for AKT1, AKT2, and AKT3 and has shown potent inhibition of AKT pathway signaling and tumor growth in mouse xenograft models explanted with cells harboring dysregulated AKT pathways^11^. It is currently in Phase IB clinical studies for treatment of certain cancers. We report encouraging results demonstrating inhibition of AKT by ARQ 092 in cells and tissues harboring AKT1 E17K mutations from patients with Proteus syndrome. These data support the clinical development of ARQ 092 in patients with Proteus syndrome, targeting this pathway as a novel treatment for this disease.

## Results

**Figure 1:**
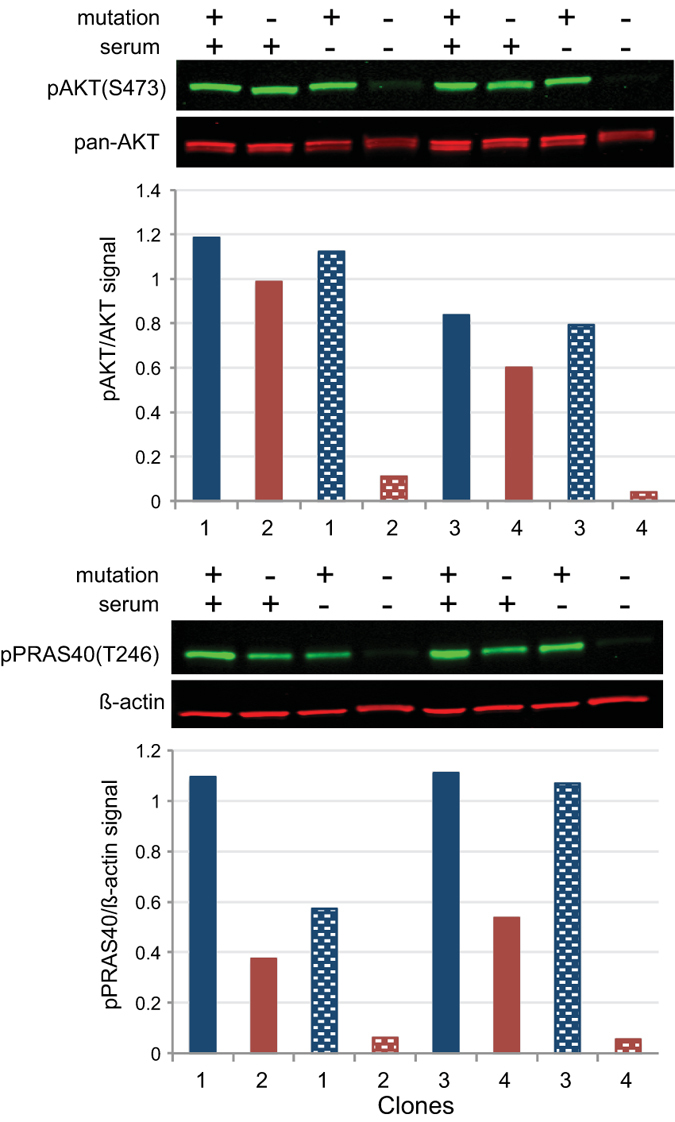
AKT remained active in mutation-positive cells in the absence of growth factors. Single cell clones isolated from fibroblasts cultured from an epidermal nevus located on the dorsum of the right hand of patient PS134 were grown with and without serum (indicated above gel images) followed by lysis and western analyses. Clones 1 and 3 (blue bars) were positive for the AKT1 E17K mutation; clones 2 and 4 (red bars) were negative. Histograms show ratios of the infrared signals from each antibody pair as described in the methods. Shaded bars represent ratios in cells grown in serum-free medium. Levels of pAKT and pPRAS40 were elevated in the absence of serum in mutation-positive cells compared to mutation-negative cells.

Previously we showed that fibroblasts positive for the AKT1 E17K mutation had elevated phospho-AKT (pAKT) levels compared to mutation-negative cells when both were grown in serum-free medium^3^. To extend these findings, pAKT levels were measured in single cell clones (SCC) that were heterozygous for the AKT1 E17K mutation or mutation-negative, and were grown in the presence or absence of serum (Fig. 1). Mutation-positive cells had markedly higher levels of pAKT than mutation-negative cells when grown without serum. PRAS40, the product of the *AKT1S1* gene that is phosphorylated at threonine 246 (T246) by AKT^12^, also showed increased phosphorylation in mutation-positive fibroblasts grown in serum-free medium. In the example shown in Fig. 1, pAKT levels were 7- to 29-fold higher and phospho-PRAS40 (pPRAS40) levels were 9- to 20-fold higher in the mutation-positive SCC grown in serum-free medium as compared to mutation-negative SCC. These differences in pAKT and pPRAS40 levels are representative of what was seen with Proteus SCC grown without serum (unpublished observations).

**Figure 2:**
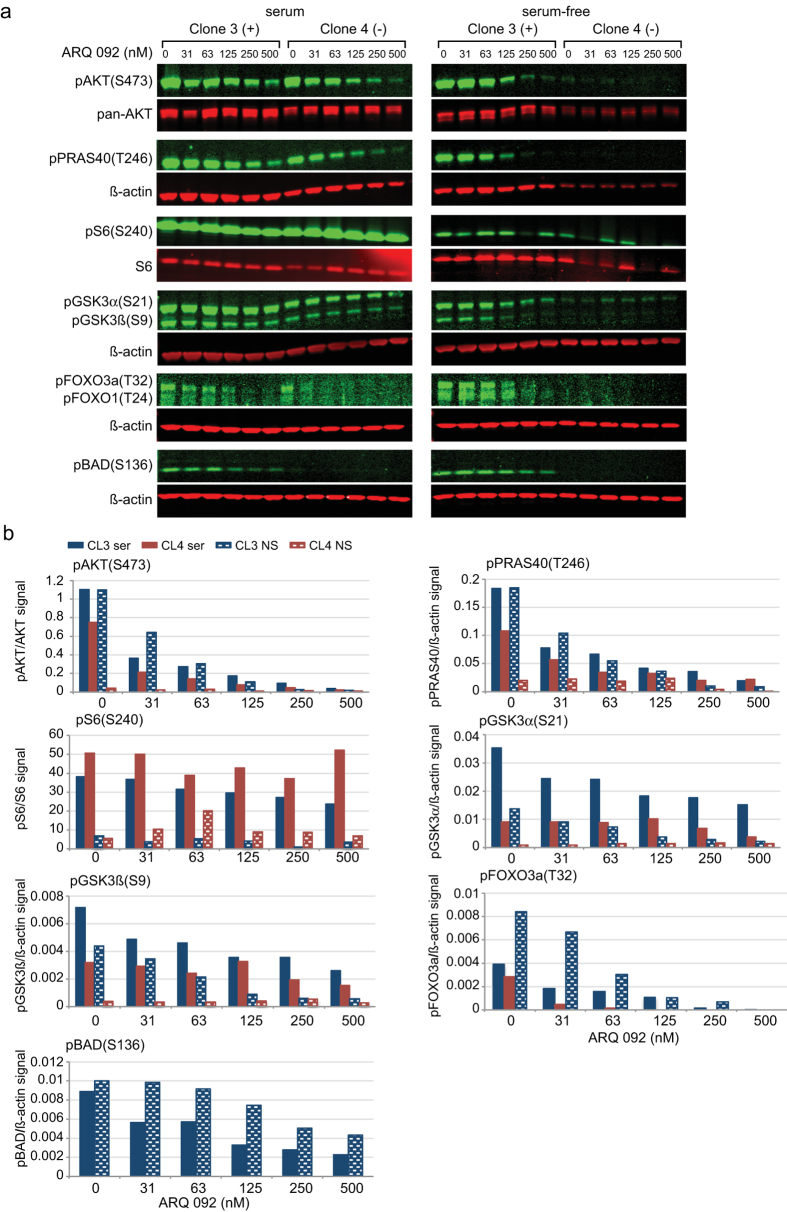
ARQ 092 decreased the level of pAKT and downstream targets in a concentration-dependent manner. Clones 3 (mutation-positive) and 4 (mutation-negative) were cultured with or without serum and in the presence of increasing concentrations of ARQ 092 as indicated. (**a**) Infrared images of western hybridizations of each clone grown using the indicated antibodies. (**b**) Histograms of the ratios of the infrared signals from each antibody pair. Levels of AKT and PRAS40 phosphorylation were reduced with increasing concentrations of ARQ 092 in both mutation-positive and negative SCC when grown with or without serum compared to levels in untreated cells. Levels of pGSK3α and pGSK3β were reduced in mutation-positive cells with ARQ 092 treatment compared to untreated cells, but were similar in all mutation-negative cells. pBAD was not detected in mutation-negative cells, but was reduced with drug treatment in mutation-positive cells compared to untreated cells. pFOXO3a was only detected in untreated mutation-positive and mutation-negative cells grown with serum and with low concentrations of ARQ 092. pFOXO1 was only detected in mutation-positive cells grown without serum and was not measured. Levels of pS6 were not consistently reduced. Results of additional experiments are shown in Supplementary Fig. 1. CL3, clone 3 (blue bars); CL4, clone 4 (red bars); ser, grown with serum (solid bars); NS, grown without serum (shaded bars).

To determine whether ARQ 092 can reduce the pAKT levels in Proteus syndrome cells, mutation-positive and -negative SCC described in Fig. 1 were incubated with increasing concentrations of ARQ 092 in the presence or absence of serum (Fig. 2 and Supplementary Fig. 1). Phosphorylation of AKT T308 and S473 was reduced in a concentration-dependent manner in both SCC grown in serum-free or serum-containing media. With as little as 30 nM of ARQ 092, pAKT levels were reduced to approximately half compared to the levels in untreated fibroblasts. With 125 nM, the AKT phosphorylation was reduced 5- to >20-fold in mutation-positive cells grown with or without serum and in mutation-negative cells grown with serum compared to levels in untreated cells. At 500 nM, all pAKT levels were nearly zero. Mutation-negative cells grown without serum showed the smallest reduction in phosphorylation as expected given that pAKT levels are very low in untreated cells. PRAS40 also showed a concentration-dependent reduction in phosphorylation at T246 with the administration of ARQ 092 to mutation-positive and negative SCC grown with or without serum compared to levels in untreated cells. Other known targets of AKT phosphorylation were also assessed^13^,^14^,^15^. Compared to untreated cells, GSK3α, GSK3β, FOXO3a and BAD all showed reduced phosphorylation in mutation-positive cells with increased concentrations of ARQ 092. In mutation-negative cells, phosphorylation of GSK3α and GSK3β was similar to untreated cells across all concentrations of ARQ 092. pBAD was undetectable in mutation-negative cells and pFOXO3a could only be measured in cells grown with serum in untreated and low concentrations of ARQ 092. Reduced phosphorylation with ARQ 092 was not consistently observed for ribosomal protein S6 (a target of AKT signaling farther downstream in the pathway^16^,^17^) at either serine 235 (S235) or serine 240 (S240) in both mutation-positive and -negative SCC. ARQ 092 did not change the levels of total AKT, PRAS40, GSK3α, GSK3β, or S6 proteins in mutation-positive and -negative SCC grown with or without serum (Supplementary Fig. 2). Reduced levels of FOXO3a were seen in mutation-positive cells grown with serum in ARQ 092-treated cells compared to untreated cells, but levels remained steady at all drug concentrations. Total BAD protein could not be detected.

In patients with Proteus syndrome, tissues are a mixture of mutation-positive and -negative cells, so we also measured AKT phosphorylation in four fibroblast cultures from the same patient that had differing levels of mutant cells. Two skin fibroblast cultures from a CCTN and one from the dorsal surface of the left hallux, and a fourth culture from apparently normal skin on the left leg were established from patient PS75. In cells used for the experiment shown in Supplementary Fig. 3a and b, the mutation levels ranged from 0–41%. Regardless of the mutation percentage, reduced AKT phosphorylation was observed with increasing concentrations of ARQ 092 in all cells and was not dependent on the serum content of the medium. The results of a replicate experiment are shown in Supplementary Fig. 3c. We conclude that ARQ 092 inhibits AKT phosphorylation and reduces pathway signaling in mutation-positive and -negative cells from patients with Proteus syndrome in a concentration-dependent manner.

**Figure 3:**
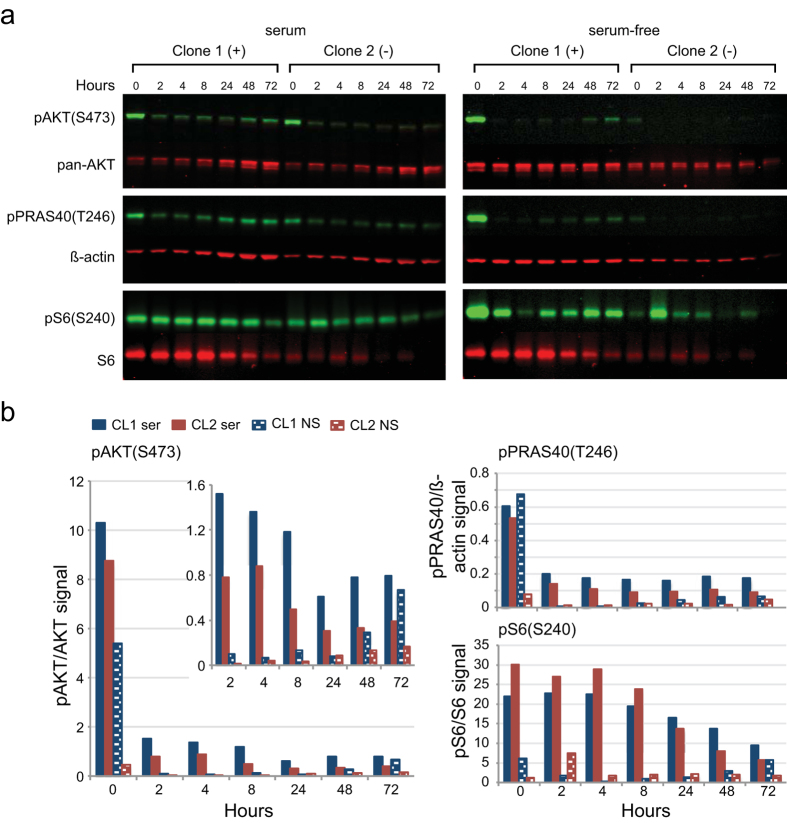
Levels of pAKT and pPRAS40 were reduced within 2 hours of treating with ARQ 092 and remained low for 72 hours. Mutation-positive (clone 1) and mutation-negative (clone 2) SCCs were cultured in the presence or absence of serum with 125 nM ARQ 092 and lysed at the times indicated. The media was not changed during the course of the experiment. (**a**) Infrared images of western hybridizations using the indicated antibodies. (**b**) Histograms of the ratios of the infrared signals for each antibody pair. The inset in the pAKT histogram shows the ratios from the ARQ 092-treated cells using a smaller scale. Note the increased signal from CL1 lysates grown in serum-free medium at 8, 48 and 72 hours. Results of additional experiments are shown in Supplementary Fig. 4. CL1, clone 1 (blue bars); CL2, clone 2 (red bars), ser, grown with serum (solid bars); NS, grown without serum (shaded bars).

To determine the time course of how ARQ 092 inhibits phosphorylation, mutation-positive and -negative SCC were treated with 125 nM ARQ 092 with or without serum. Fibroblasts were collected at several time points from two to 72 hours (Fig. 3 and Supplementary Fig. 4). The media was not changed for the duration of the study. By 2 hours, pAKT levels dropped 7- to >50-fold compared to baseline, and they remained low for the duration of the experiment. However, in un-stimulated mutant cells, pAKT levels started to rise at later time points suggesting that in culture, mutant fibroblasts may need to be treated with drug more frequently to suppress AKT signaling. PRAS40 showed a similar pattern of phosphorylation whereas phosphorylation of S6 showed a more modest reduction compared to untreated cells.

**Figure 4:**
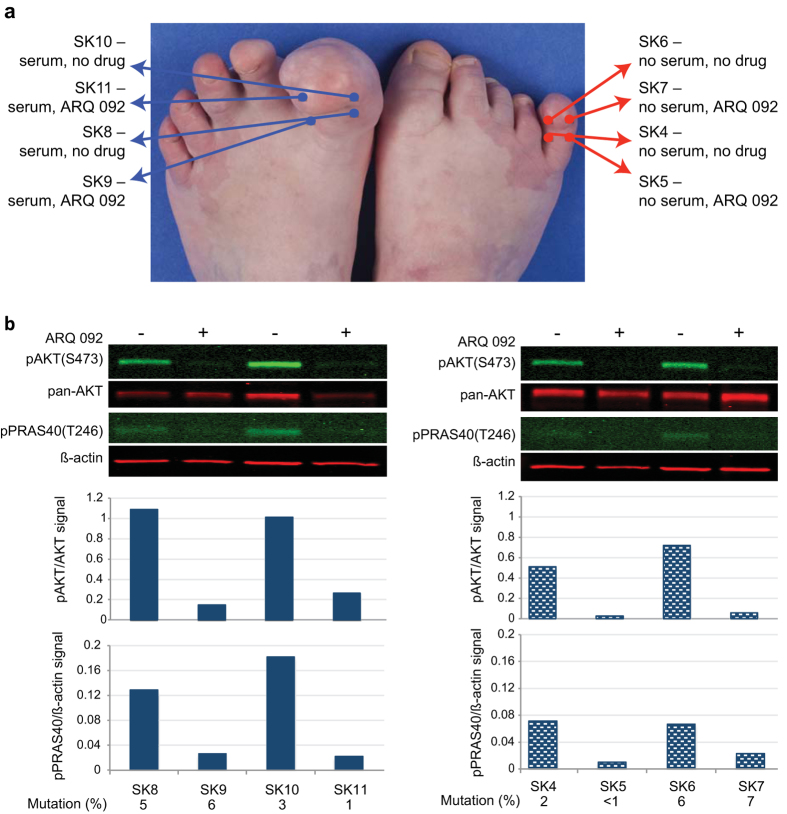
ARQ 092 decreased phosphorylation of AKT and PRAS40 in tissues from a patient with Proteus syndrome. (**a**) The location and incubation conditions of punch biopsies taken from two affected toes from patient PS95 are indicated on the photo. Proteins and DNA were extracted as described in Methods. (**b**) Infrared images of western hybridizations using the indicated antibodies. Histograms below each lane represent the ratio of indicated antibody pair in each lysate. Symbols above images indicate the presence or absence of ARQ 092. *AKT1* E17K mutation levels for each biopsy are shown below the graphs.

Tissue pAKT levels were measured in eight, three-millimeter skin biopsies from two affected toes of patient PS95 that were incubated in DMEM for 96 hours at 37 °C with or without serum and 125 nM ARQ 092 (Fig. 4). Phosphorylation of AKT was reduced from four- to sevenfold for tissues incubated with serum and nine to 27-fold for those in serum-free medium when ARQ 092 was added. pPRAS40 levels were also reduced though not as dramatically as pAKT. From these results, we conclude that we will be able to assess the pharmacodynamic effect of ARQ 092 on AKT signaling in patients treated with the drug by measuring pAKT levels in tissue biopsies.

**Figure 5:**
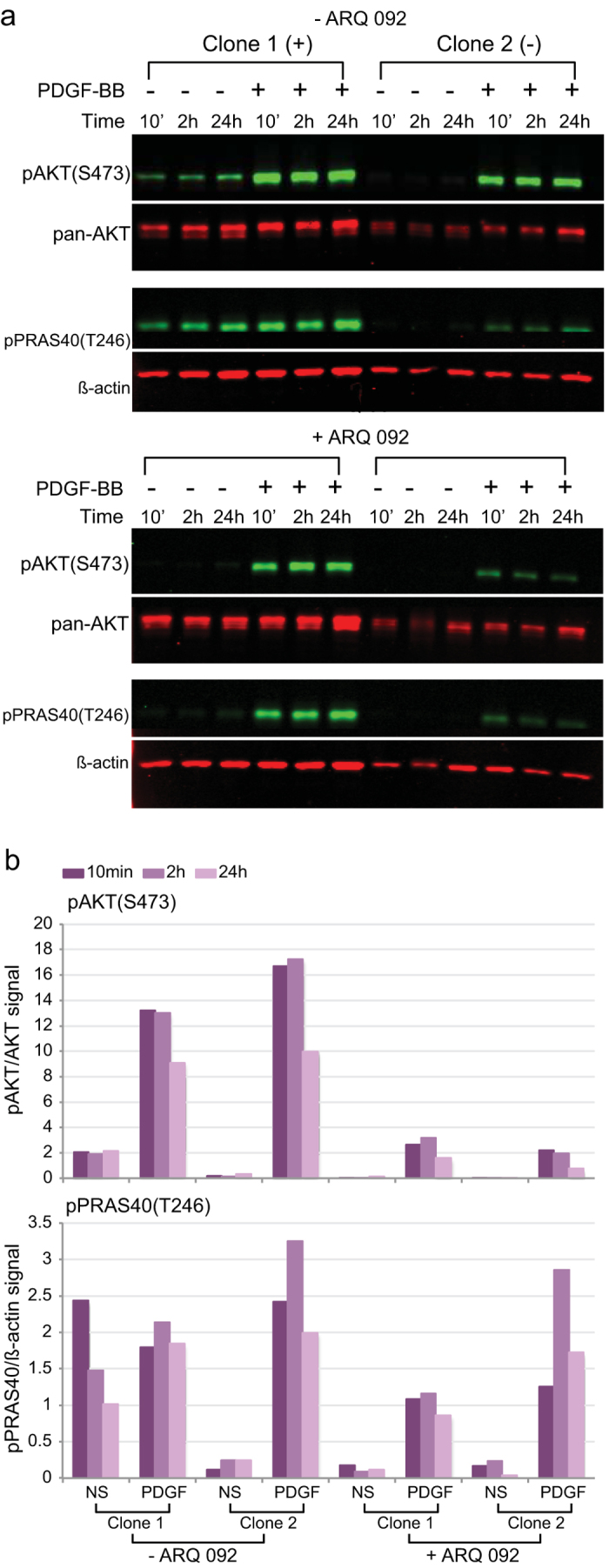
Platelet-derived growth factor-BB (PDGF-BB) stimulation in cells treated with ARQ 092 resulted in elevated levels of pAKT and pPRAS40. Mutation-positive (clone 1) and negative (clone 2) SCC were serum starved, treated +/−ARQ 092 and stimulated with PDFG-BB as described in the Methods. Cells were collected at 10 minutes, 2 hours, and 24 hours after PDGF-BB addition. (**a**) Infrared images of western hybridizations using the indicated antibodies. (**b**) Histograms of the ratios of the infrared signals for each antibody pair. PDGF-BB stimulation resulted in a 12- to 70-fold increase in pAKT in ARQ 092-treated clone 1 and 24- to 50-fold increase in ARQ 092-treated clone 2 compared to un-stimulated cells. In untreated SCC, pAKT levels increased four- to sevenfold in clone 1 and 30– to >100 fold for clone 2 with PDGF-BB stimulation. For pPRAS40, PDGF-BB stimulation resulted in a 6– to 12-fold increase ARQ 092-treated clone 1 and 7– to 50-fold increase in ARQ 092-treated clone 2 compared to un-stimulated cells. In untreated SCC, pPRAS40 levels increased 8- to 20-fold in clone 2 and showed a 30% decrease or twofold increase in clone 1 upon PDGF-BB stimulation. Taken together, these results showed that Proteus SCCs are able to respond to PDGF-BB stimulation by increasing pAKT and pPRAS40 levels even when AKT signaling is suppressed by ARQ 092. Results of an additional experiment are shown in Supplementary Fig. 4. NS, grown without serum; PDGF, PDGF-BB stimulated

As discussed earlier, the goal for treatment of patients with Proteus syndrome is to reduce AKT signaling without disrupting all cellular function associated with this pathway. To determine if cells could respond to growth factor stimuli in the presence of ARQ 092, we measured pAKT and pPRAS40 levels in cells that had been serum-starved and then stimulated by platelet derived growth factor – BB (PDGF-BB) in the presence or absence of 125 nM ARQ 092 (Fig. 5 and Supplementary Fig. 5). PDGFs have growth promoting activities in fibroblasts^18^, have been implicated in the development of multiple organ systems and several diseases^19^, and activate AKT^20^. Mutation-positive and -negative cells responded rapidly as shown by the dramatic increase in pAKT levels compared to levels in un-stimulated cells within 10 minutes of PDGF-BB addition. While the magnitude of the phosphorylation was lower in all cells treated with ARQ 092, the pattern of the response was very similar. Both mutation-positive and -negative SCC showed a 12- to 70-fold increase in pAKT levels upon addition of PDGF-BB when they were treated with ARQ 092 compared to un-stimulated drug-treated SCC. This is similar to the 30- to >100-fold increase in pAKT when mutation-negative SCC grown without ARQ 092 were stimulated. Untreated mutation-positive SCC only had a four- to sevenfold increase in pAKT upon PDGF-BB-stimulation, however, the pAKT levels for the un-stimulated mutation-positive SCC were already 7- to 17-fold higher than those of the un-stimulated mutation-negative SCC. pPRAS40 levels were also elevated in ARQ 092-treated, PDGF-BB-stimulated, SCC as compared to those that remained in serum-free medium, but in contrast to pAKT, levels of pPRAS40 in ARQ 092-treated cells, PDGF-BB-stimulated cells were similar to, or above, those in PDGF-BB-stimulated cells not treated with ARQ 092, suggesting that PDGF-BB can activate PRAS40 independent of AKT. When cells were stimulated with serum instead of PDGF-BB, similar results were seen in ARQ 092-treated SCC. (Supplementary Fig. 6). We hypothesize that for normal growth and development, inhibition of AKT phosphorylation will need to be relaxed periodically in response to stimuli. We conclude that while 125 nM of ARQ 092 represses nearly all AKT phosphorylation in Proteus syndrome cells, they remain able to modulate signaling in response to growth factor stimulation.

**Figure 6:**
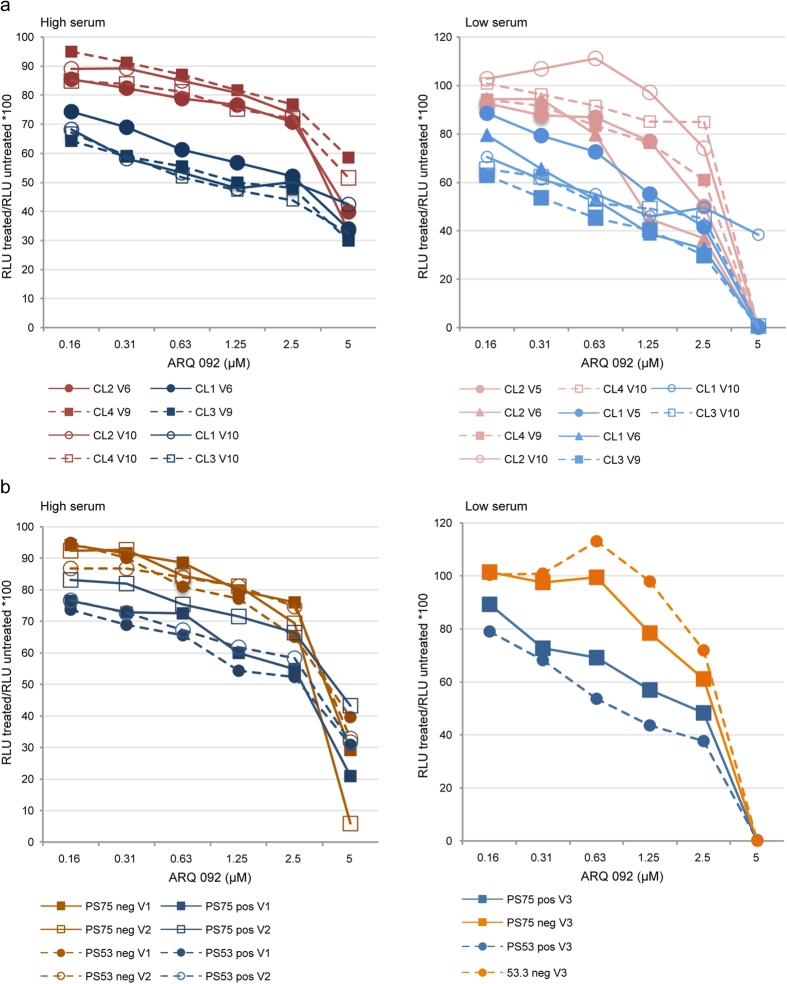
High concentrations of ARQ 092 were needed to reduce viability of cells from patients with Proteus syndrome. ATP levels were measured using a luminescent assay in mutation-positive and mutation-negative SCCs and patient fibroblasts grown in high (10%) or low (0.5%) serum. The percentage of viable cells at a given dose was calculated as described in the Methods. (**a**) Luminescence ratios of SCCs grown in high or low serum with increasing concentrations of ARQ 092. Clones 1 and 3 are mutation-positive; clones 2 and 4 are mutation-negative. (**b**) Luminescence ratios of mutation-positive and mutation-negative fibroblasts from patients PS53 and PS75 grown in high or low serum with increasing concentrations of ARQ 092. The level of the AKT1 E17K mutation in cells from patients PS53 and PS75 were 37–42% and zero in the PS53 and PS75 mutation-negative cells. Mutation-positive clones and cells had lower viability and were clearly separated from mutation-negative clones and cells in both high and low serum. At 1.25 uM, levels 10-fold higher than necessary to effectively reduce AKT signaling, 40–70% of cells were still viable. CL1, clone 1; CL2, clone 2; CL3, clone 3; CL4, clone 4; pos, mutation-positive cells; neg, mutation negative cells V-numbers in the legend indicate the experiment number for the data represented by that line.

Because the goal of this study is to discover potential therapeutic agents that will reduce AKT signaling in Proteus cells without killing them, we wanted to determine what levels of ARQ 092 could be tolerated before becoming cytotoxic. We measured ATP levels using a luminescent assay^21^ in SCC and cultures with varying numbers of mutation-positive cells that were grown for 72 hours with increasing concentrations of ARQ 092 (Fig. 6). In both high (10%) and low (0.5%) serum, the mutation-positive cells were more sensitive to increased levels of ARQ 092 than mutation-negative cells. However, even at 1.25 μM, a level 10-fold higher than needed to effectively reduce AKT signaling, 40–70% of mutant cells were still viable. At 5 μM ARQ 092, the percentage of viable cells grown in low serum was zero in nearly every experiment suggesting that the un-stimulated cells were more sensitive to the drug. These results were replicated when cells were treated with ARQ 092 and assayed by manual cell counting (Supplementary Fig. 7). We conclude that at levels sufficient to suppress AKT signaling, ARQ 092 will cause little reduction in cell viability.

## Discussion

In this study, we evaluated the potential utility of ARQ 092 as a pharmacologic therapy for patients with Proteus syndrome. The treatment objectives for Proteus syndrome are distinct from those for most types of cancer. While both cancer and Proteus syndrome are mosaic disorders, the consequences and dysfunction of the cells have different clinical implications. In cancer, the objective is generally to kill or eliminate malignant cells from the patient. In an overgrowth disorder like Proteus syndrome, the objective is to eliminate or reduce the abnormal constitutive signal caused by the mutation to normalize cellular functions and reduce overgrowth. Because patients with Proteus syndrome can have high levels of mutant cells, cellular toxicity is highly undesirable. To evaluate a targeted therapeutic for Proteus syndrome, we used an *in vitro* model based on cells cultured from patients with Proteus syndrome, as there is no animal model for the AKT1 E17K mutation. We used both AKT1 E17K mutation-positive and mutation-negative SCC cells and fibroblast cultures with heterogeneous mutation levels, all derived from patients with Proteus syndrome. ARQ 092 is an orally available, selective, and potent pan-AKT inhibitor currently in early clinical development for oncology, with IC_50_ values in the low nanomolar range for AKT1, AKT2, and AKT3. Kinase profiling showed that ARQ 092 had IC_50_ values less than 1 μM against five other kinases but with far lower potency (26 to 162 fold) than it had against AKT1. ARQ 092 inhibits pAKT and its downstream target, pPRAS40, in AN3CA cells with IC_50_ of 62 nM for pAKT(T308), 40 nM for pAKT(S473), and 312 nM for pPRAS40(T246)^11^. Based on these results from malignant cells, we set out to evaluate it as a candidate therapeutic for Proteus syndrome.

Because Proteus syndrome is a mosaic disorder, patients typically have a mix of AKT mutation-positive and negative cells in affected areas. This results in varying levels of the mutation in tissues. To date we have isolated over 35 positive SCC and have tested hundreds of cells and tissues from over 50 patients and never seen mutation levels over 50%. This indicates that the AKT1 E17K mutation exists in a heterozygous, mosaic state in patients with Proteus syndrome and that at the cellular level the phenotype is a combination of the activity of both alleles. Currently, no *in vivo* models exist for Proteus syndrome to evaluate new therapeutics hence we developed patient-derived specimens to assess PI3K/AKT pathway inhibitors in this disease. This approach of relying on *in vitro* cellular models is supported by *in vivo* data from the ongoing development of ARQ 092 in cancer. Preclinical *in vivo* and *in vitro* cancer models have demonstrated that ARQ 092 targets the PI3K/AKT pathway and AKT specifically^11^,^22^.

Starting with SCC, these data show that ARQ 092 reduces pAKT levels and pPRAS40 in both mutant and wild type cells. In mutated cells, ARQ 092 inhibits phosphorylation in a concentration-dependent manner. These lower pAKT and pPRAS40 levels were similar to those in untreated wild type fibroblasts but did not completely extinguish pathway signaling. Similar pathway knockdown results have been shown in bladder cancer cells (KU-19-19) harboring the AKT1 E17K mutation as well as in other cancer cells that do not contain this mutation (e.g., MDA-MB-453 cells)^11^.

We also explored the effect of ARQ 092 on patient-derived cell cultures and tissue with naturally occurring levels of mutated cells. Again, AKT phosphorylation was markedly reduced in a concentration-dependent manner, with and without serum, in both cell cultures and tissue regardless of mutation levels. As anticipated, pPRAS40 levels were also reduced although not as dramatically as was pAKT. These results corroborate the findings with the SCC and indicate that ARQ 092 can penetrate into multiple layers of cells and will inhibit AKT signaling independent of the degree of mutated cells present.

In time course experiments, ARQ 092 was shown to inhibit phosphorylation rapidly in Proteus syndrome cells. When mutation-positive and negative SCC were exposed to ARQ 092 with or without serum, within 2 hours pAKT levels were very low in both the mutation-positive and negative Proteus syndrome fibroblasts. Of note, in un-stimulated mutant cells, pAKT levels started to rise by 8 hours suggesting that it would be best to dose ARQ 092 frequently to maintain inhibition of AKT1 E17K in patients with Proteus syndrome. These data also suggest that any toxic effects of pAKT reduction should be promptly reversible by discontinuing treatment. Again, we have as an explicit goal no cellular toxicity and a desire to maintain normal growth factor receptor signaling through this pathway. To verify that the PI3K/AKT pathway was still functional in the presence of ARQ 092, we stimulated serum-starved mutant and wild type cells with and without ARQ 092. By measuring the pAKT and pPRAS40 levels, we conclude that although ARQ 092 exposed cells did have reduced phosphorylation, they retained the ability to generate a pAKT response to growth factors in both mutant and wild type cells. Hence, to maintain pathway inhibition and minimize toxicities shown to occur with AKT inhibitors when the pathway is strongly inhibited^23^, we anticipate that a tissue level of no more than 125 nM of ARQ 092 should be sufficient to accomplish this objective in patients with Proteus syndrome.

In phase I clinical studies with ARQ 092 in cancer patients (n = 3), mean steady state C_max_ plasma levels of 142 nM were achieved with 20 mg QD dosing with only one drug-related adverse event of Grade 2 diarrhea. At a dose of 60 mg QD, the maximum tolerated dose (MTD) in patients with cancer (n = 6), resulted in mean steady state C_max_ plasma levels of 497 nM^22^. We conclude that doses of ARQ 092 significantly lower than the MTD for cancer will be sufficient to treat patients with Proteus syndrome. These are encouraging results because we anticipate that long-term treatment (e.g., decades) may be necessary for patients with Proteus syndrome, based on the natural history of the disease which causes the most overgrowth in the first two decades of life^1^.

We also measured additional downstream effectors in the PI3K/AKT pathway to better understand the effects of ARQ 092 on the PI3K/AKT pathway in Proteus syndrome cells. We found that ARQ 092 reduced phosphorylation of GSK3α and GSK3β in mutation-positive cells whereas in mutation-negative cells, phosphorylation levels in treated and untreated cells were similar. pBAD was only detected in mutation-positive cells suggesting that the AKT1 E17K mutation is causing aberrant activation of this protein. This increased activation was reduced by ARQ 092 treatment. Interestingly, we found that ribosomal protein S6, a downstream target of AKT, showed little to no reduction in phosphorylation with ARQ 092 suggesting that at these concentrations, ARQ 092 will not have a generalized inhibition of protein synthesis.

We also determined the levels of ARQ 092 required to reduce cell viability in Proteus syndrome cells. These experiments were performed to evaluate our treatment objective of inhibiting signaling in mutant cells without engendering large-scale cell or tissue toxicity. In both high and low serum, the mutation-positive cells were more sensitive to increased levels of ARQ 092 than mutation-negative cells. However, even at levels 10-fold higher than needed to effectively reduce AKT signaling, 40–70% of mutant cells were still viable. Only at concentrations as high as 5,000 nM were most cells killed. These data indicate that a dosing schedule of ARQ 092 that effectively suppresses AKT signaling without loss of viability is possible.

In conclusion, we have shown that AKT1 E17K-positive cell lines and tissues derived from patients with Proteus syndrome respond in a concentration-dependent manner to ARQ 092 and that ARQ 092 offers the potential to reduce AKT signaling at concentrations predicted to have few clinical adverse effects, thus validating ARQ 092 as a potential novel therapeutic for Proteus syndrome and supporting its clinical development in patients with Proteus syndrome.

## Methods

### Sample acquisition

This study was carried out in accordance with relevant guidelines and regulations after being reviewed and approved by the Institutional Review Board of the National Human Genome Research Institute (94-HG-0132). Samples were obtained from research participants with Proteus syndrome after written informed consent was obtained. Fibroblast cultures were established as described previously^24^. AKT1 E17K mutation-positive and -negative single cell clones (SCC) were isolated by limiting dilution from fibroblasts obtained from an epidermal nevus located on the dorsum of the right hand of a patient with Proteus syndrome (PS134).

### Cell culture

Fibroblasts were plated at equal densities in Dulbecco’s Modified Eagle’s Medium (DMEM) containing 10% fetal bovine serum. For studies of quiescent cells, fibroblasts were transferred to serum-free medium for 20–24 hours prior to drug treatment or cell lysis. Fibroblasts were incubated for 20–24 hours with drug in medium with or without serum before cell lysis unless otherwise indicated. ARQ 092 was kindly provided by ArQule, Inc (Burlington, MA, USA).

### Growth factor stimulation

Cells were grown in serum-free medium for 20–24 hours. Half of the cells were transferred to serum-free medium containing 125 nM ARQ 092 and incubated for 2 hours. Cells were then switched to serum-free medium containing 30 ng/ml platelet-derived growth factor-BB (PDGF-BB, product #100-14B, Peprotech, Inc. Rocky Hill, NJ, USA) or medium containing 10% fetal bovine serum, +/−125 nM ARQ 092 followed by cell lysis at times indicated. Proteins were extracted after washing the cells with PBS, adding 1× InstantOne ELISA Cell Lysis Mix (eBioscience, San Diego, CA, USA), shaking the plates on an orbital shaker for 10 min, and then passing the lysates through a 1 cc syringe with a 25 gauge needle 4–5 times.

### Tissue drug studies

Skin biopsies (3 mm) were incubated in 50 ml conical tubes with 5 ml of DMEM with or without serum and 125 nM ARQ 092 for 96 hours at 37 °C. Tissues were frozen on dry ice and stored at −80 °C for 2 days. Each biopsy was dissected such that protein was extracted from approximately 2/3 and DNA was extracted from the remainder. Protein was isolated by homogenizing the tissue in 500 μl 1× cell lysis buffer (Cell Signaling Technologies, Danvers, MA, USA) supplemented with HALT inhibitors (Thermo Scientific, Waltham, MA, USA) using a TissueRupter (Qiagen, Valencia, CA, USA) for 2 minutes on ice. Lysates were vortexed for 10 minutes followed by centrifugation for 10 minutes at 21,000 rcf at 4 °C. The cleared lysate was transferred to a new tube and frozen at −80 °C. DNA was extracted by standard methods and mutation levels were measured by a custom restriction fragment length polymorphism assay as described^3^.

### Western analyses

Western analyses were performed as described^25^. For each experiment, lysates from a cell line grown with or without serum were run on the same gel and all gels were run at the same time. Two gels were transferred to a single membrane. If more than one membrane was used for an experiment, a single solution for each primary and secondary antibody pair was made and divided between the two membranes that were subsequently hybridized in separate trays. Antibodies used included pAKT(S473) (product #4060), pAKT(T308) (product #2965); pan-AKT (product #2920 and 4691), pPRAS40 (product #2997), PRAS40 (product # 2610), pS6(S235) (product #2211), pS6(S240) (product #5364) S6 (product #2317 and 2217), pFOXO1/FOXO3a (product #9464), FOXO3a (product #2497), pGSK3α/β (product #9331), GSK3α/β (product #5676), pBAD (product #4366) and BAD (product #9239) from Cell Signaling Technologies (Danvers, MA, USA) or β-actin (product #A5441) from Sigma-Aldrich (St. Louis, MO, USA). Membranes were scanned and intensities calculated using a semi-automated infrared imaging system (Odyssey scanner and Image Studio Lite software, LiCor BioSciences Inc., Lincoln, NE, USA). Histograms show ratios of infrared signal intensities of indicated antibodies. For experiments using two membranes, histograms of infrared signal ratios graphed by membrane are shown in Supplementary Fig. 8.

### Viability assays

Cells were plated in 96-well dishes in DMEM containing 10% serum. The next day the cells were washed with PBS and re-fed with DMEM containing either 10% (high) or 0.5% (low) serum. After 24 hours, triplicate wells were re-fed with DMEM containing high or low serum and the indicated concentration of drug. After 72 hours, viability was measured using the CellTiter-Glo^®^ assay (Promega, Madison, WI, USA). Luminescence was averaged for the triplicate wells. Percent viability for each drug concentration was calculated by: average relative luminescence units (RLU) treated cells/average RLU untreated cells ×100. For viability measures based on cell counts, cells were plated in 24-well dishes and grown 1–4 days. Cells were washed, re-fed and treated as described above using duplicate wells. After 72 hour hours cells were detached with trypsin and manually counted. Percent viability for each drug concentrated was calculated by: average number of cells treated/average number of cells untreated ×100.

## Additional Information

**How to cite this article**: Lindhurst, M. J. *et al.* Repression of AKT signaling by ARQ 092 in cells and tissues from patients with Proteus syndrome. *Sci. Rep.* **5**, 17162; doi: 10.1038/srep17162 (2015).

## Supplementary Material

Supplementary Information
