# Supplementary material for: Repression of AKT signaling by ARQ 092 in cells and tissues from patients with Proteus syndrome
Source: Sci Rep. 2015 Dec 11;5:17162. doi: 10.1038/srep17162 (PMC4675973; doi:10.1038/srep17162)
Supplement: Supplementary Information [file srep17162-s1.pdf]

Supplementary Data

Repression of AKT signaling by ARQ 092 in cells and tissues from patients with Proteus syndrome.

Marjorie J. Lindhurst[1*], Miranda R. Yourick[1], Yi Yu[2], Ronald E. Savage[2], Dora Ferrari[2], Leslie G. Biesecker[1]

[1]National Human Genome Research Institute, National Institutes of Health, Bethesda, MD, USA, [2]ArQule, Inc., Burlington, MA, USA

Supplementary Figure 1

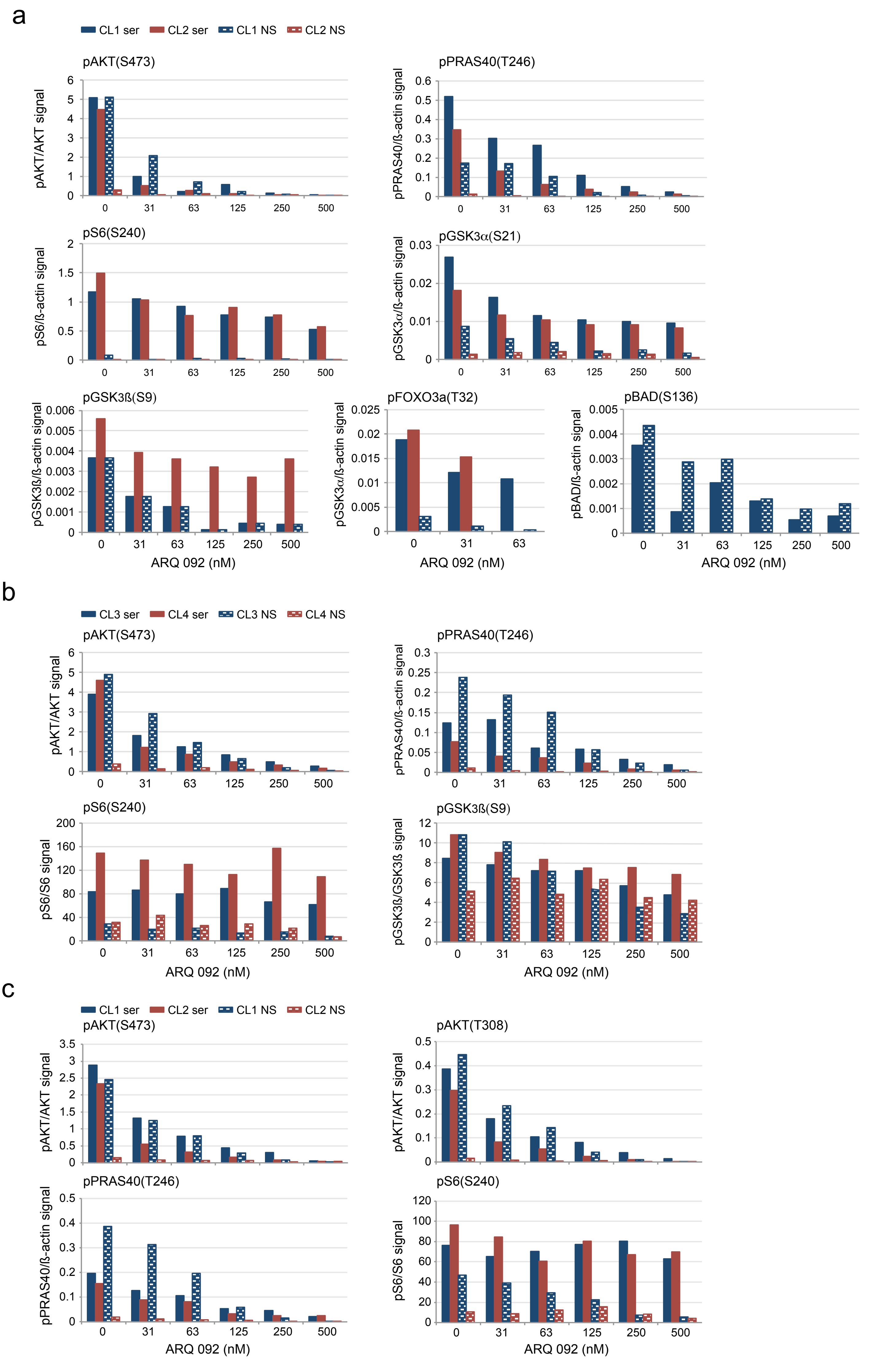

Supplementary Fig. 1. Additional experiments (a, b and c) showed that ARQ 092 reduced phosphorylation of AKT and downstream targets compared to untreated cells. Experiments were performed as described for Fig. 2. Histograms depict ratios of signals from the antibody pairs. CL1, clone 1 and CL3, clone 3 are mutation-positive (blue bars); CL2, clone 2 and CL4, clone 4 are mutation-negative (red bars); ser, grown with serum (solid bars); NS, grown without serum (shaded bars)

Supplementary Figure 2

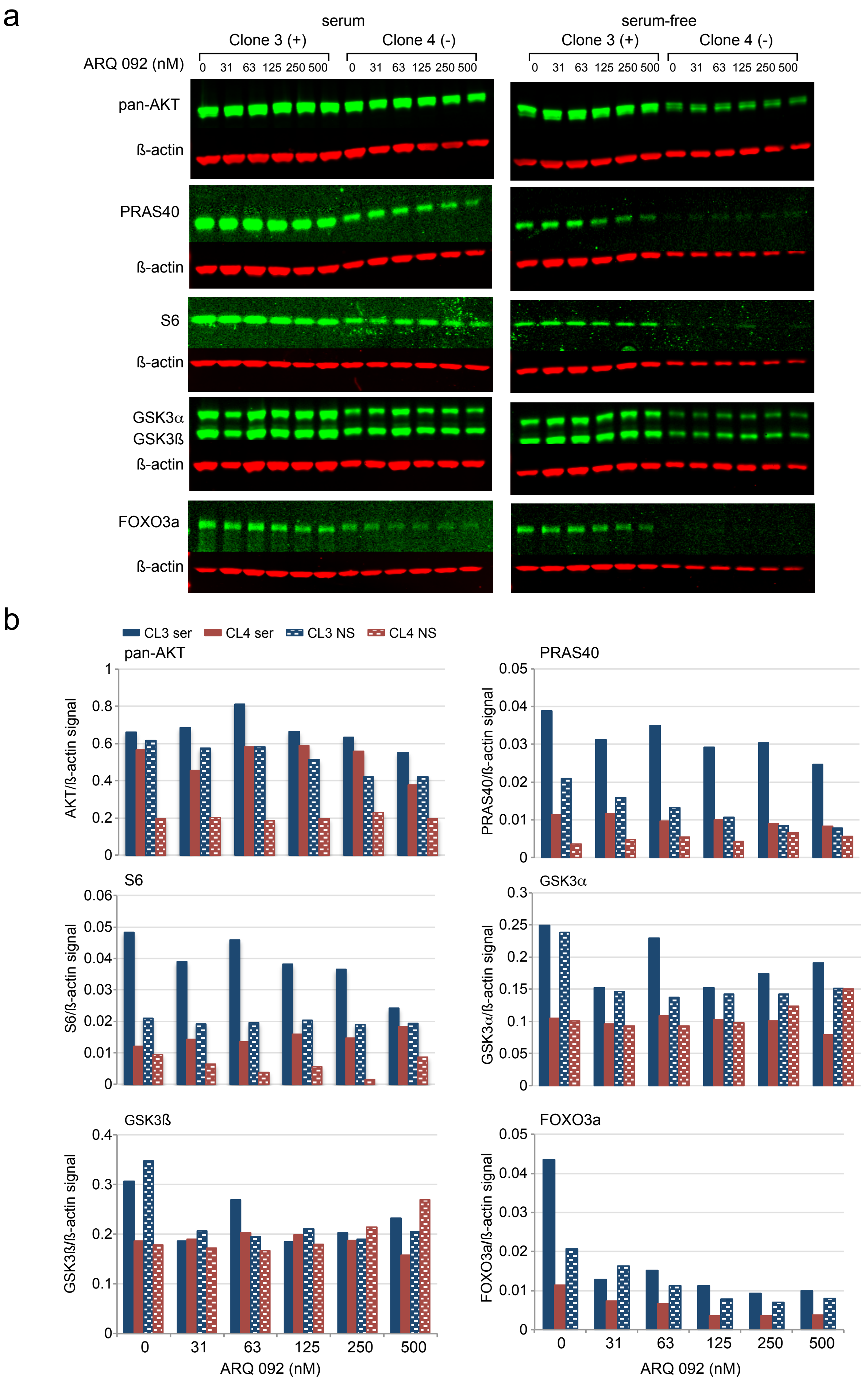

c

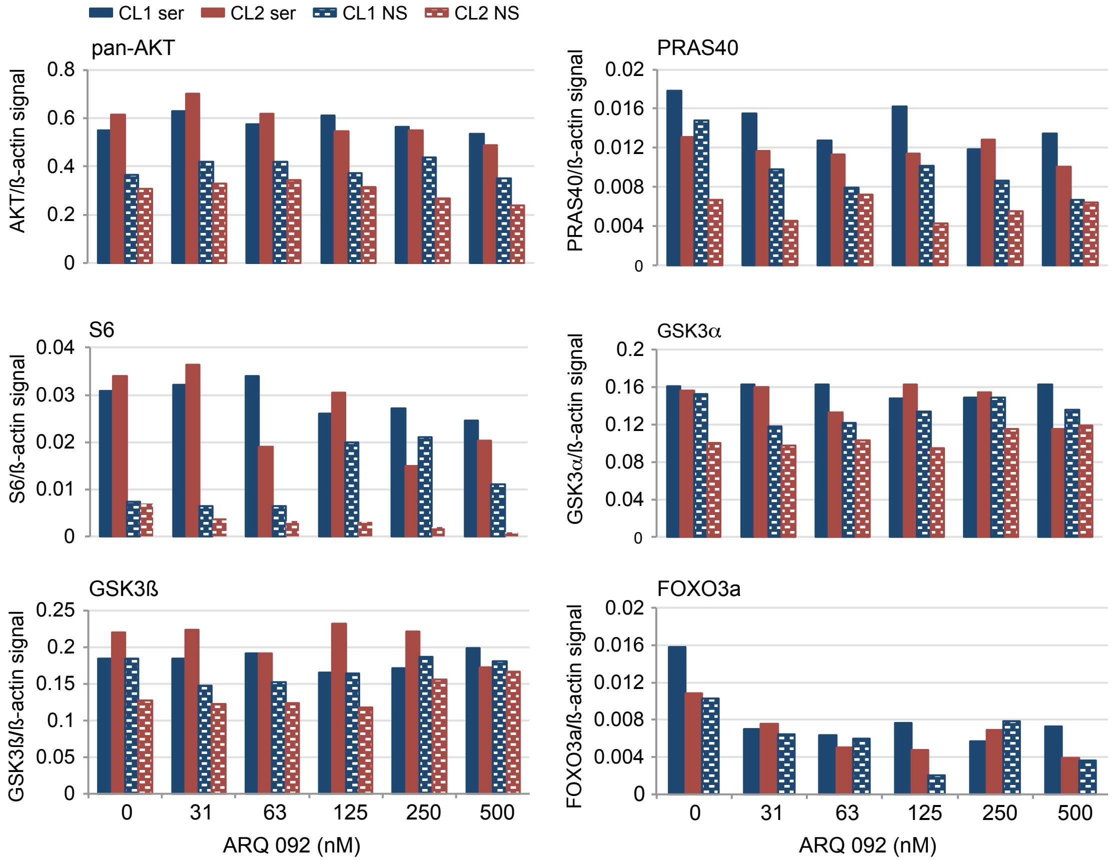

d

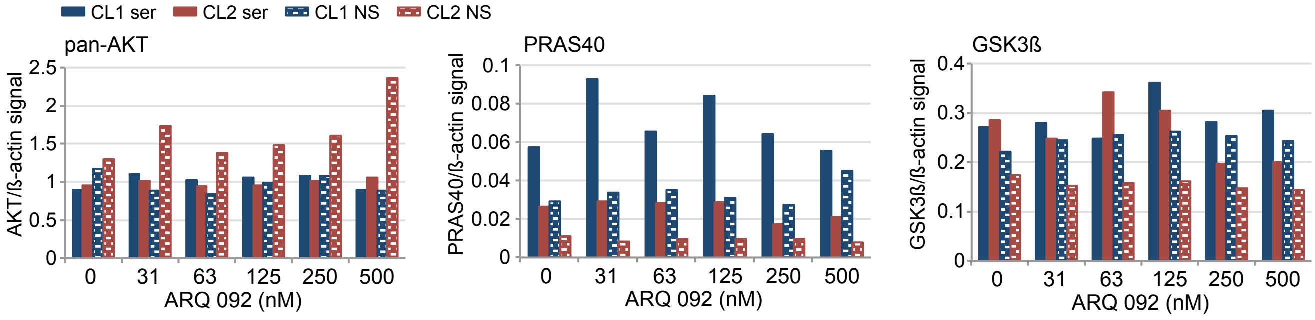

Supplementary Fig. 2. ARQ 092 did not reduce levels of total AKT protein. Lysates from experiments depicted in Fig. 2 and Supplementary Fig. 1 were assayed for levels of total AKT and downstream targets using the antibodies indicated. a) Infrared images of western hybridizations from lysates used in the experiment shown in Fig 2. b) Histograms of the ratio of the infrared signal from each antibody pair shown in a. c and d) Histograms of the ratio of the infrared signal of the indicated antibodies using lysates from the experiments shown in Supplementary figure 1a and b. Little change was seen in the level of the proteins assayed in ARQ 092-treated cells compared to untreated cells except with FOXO3a in mutation-positive cells grown with serum. CL1, clone 1 and CL3, clone 3 are mutation-positive (blue bars); CL2, clone 2 and CL4, clone 4 are mutation-negative (red bars); ser, grown with serum (solid bars); NS, grown without serum (shaded bars)

Supplementary Figure 3

a

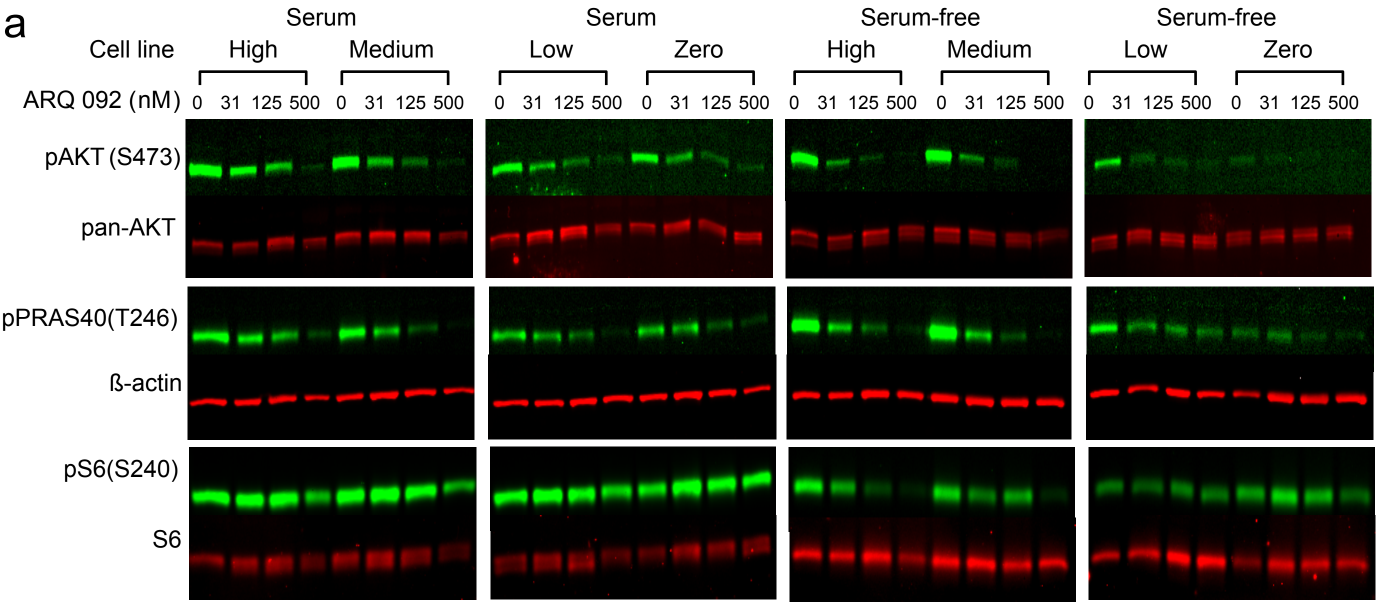

b

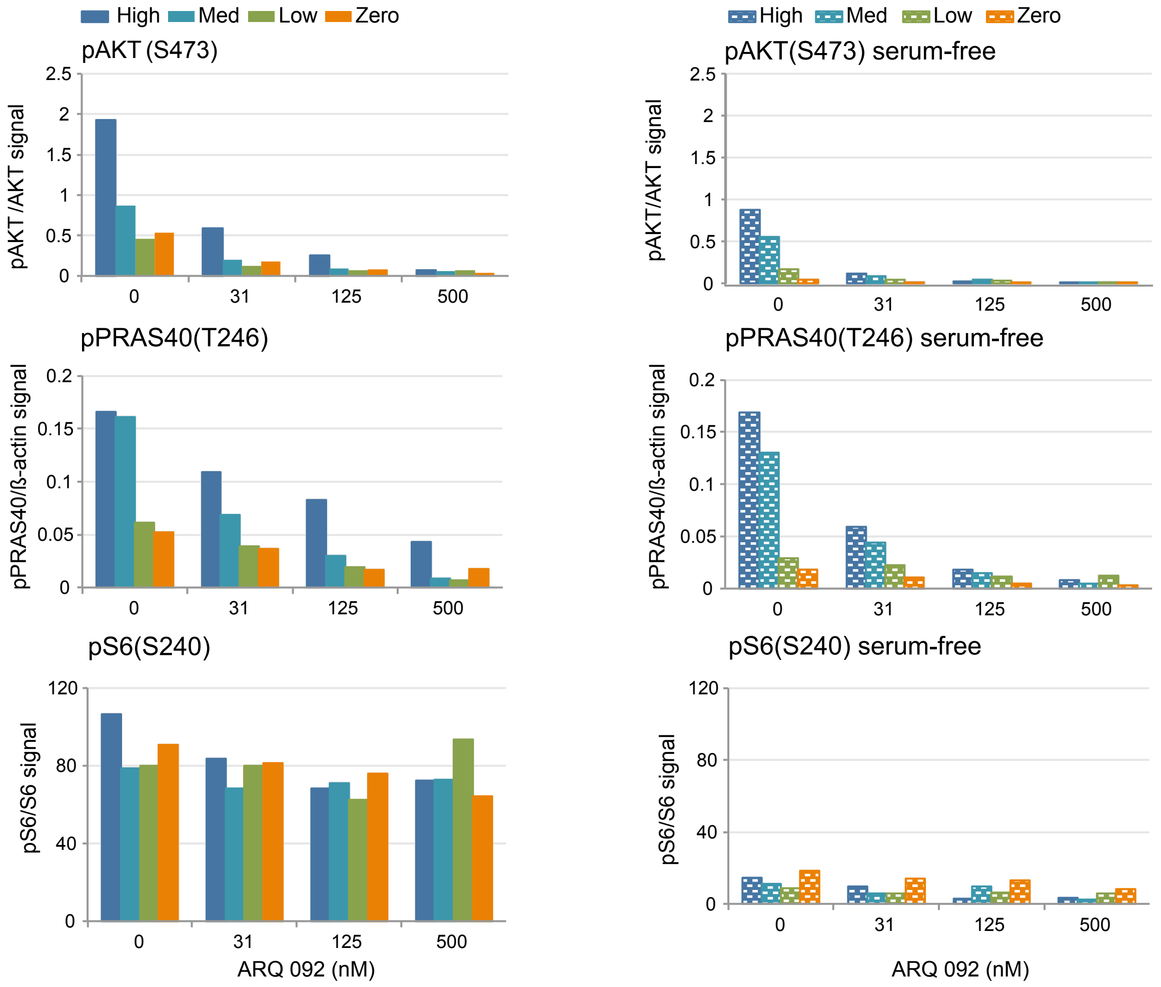

C

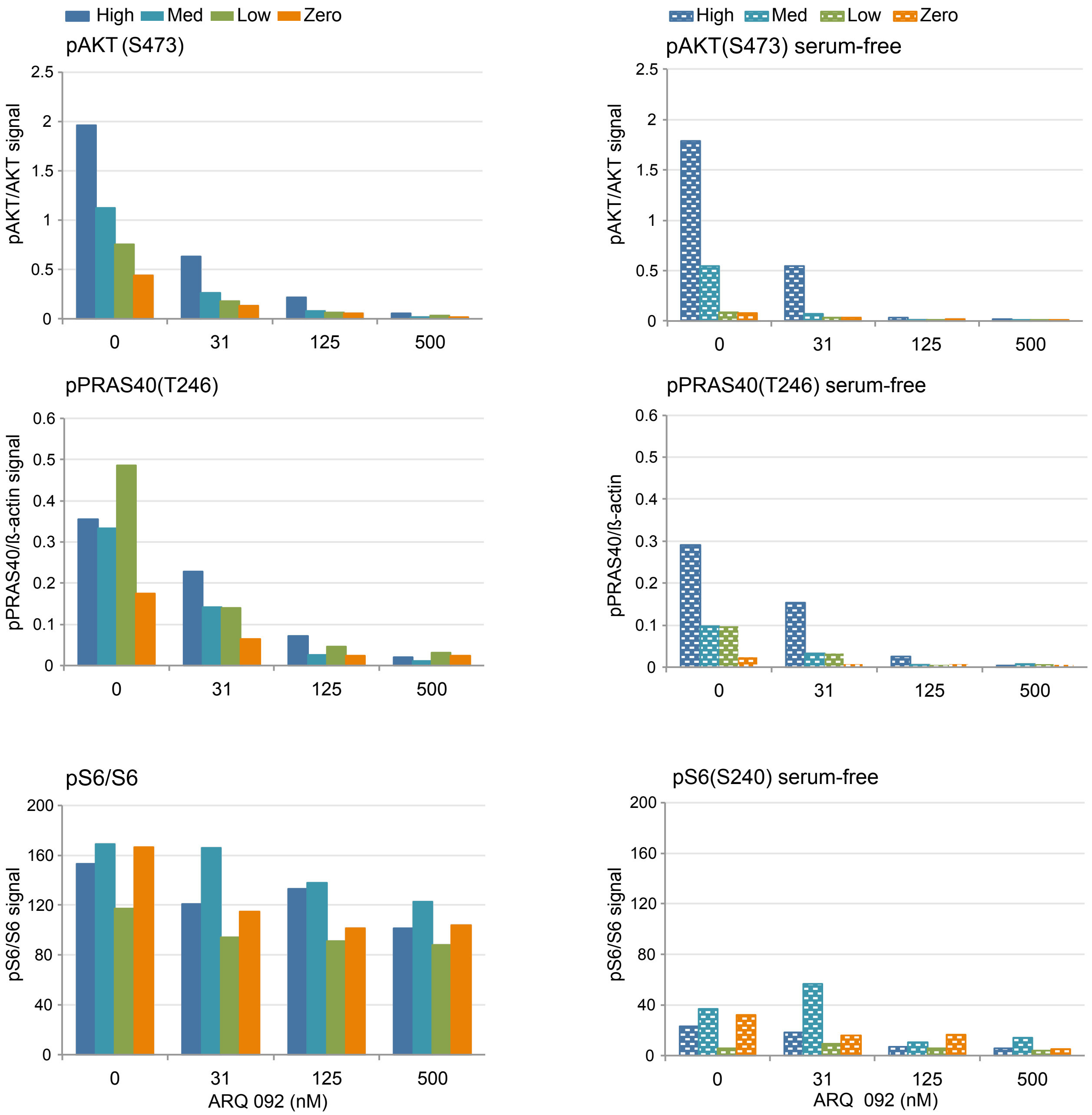

Supplementary Fig. 3. ARQ 092 decreased pAKT and pPRAS40 levels in fibroblasts with varying numbers of mutation-positive cells. a) Western analyses of protein lysates from four different fibroblast cultures from patient PS75 that were grown in the presence or absence of serum and with the indicated concentrations of ARQ 092. b) Ratios of infrared signals of indicated proteins extracted from western analyses in panel a. Ratios of antibody pairs from cells grown in normal media are graphed on the left; ratios of antibody pairs from cells grown without serum are graphed on the right using the same scale. *AKT1* c.49G>A p.Gu17Lys levels were High, 41%; Medium, 14%; Low, 3% and Zero, 0% for this experiment. C) Results from a second experiment with the same PS75 fibroblast cultures treated and analyzed as described in a. Mutation levels were High, 32%; Medium, 4%; Low, 2% and Zero, 0% for this experiment.

Supplementary Figure 4

a

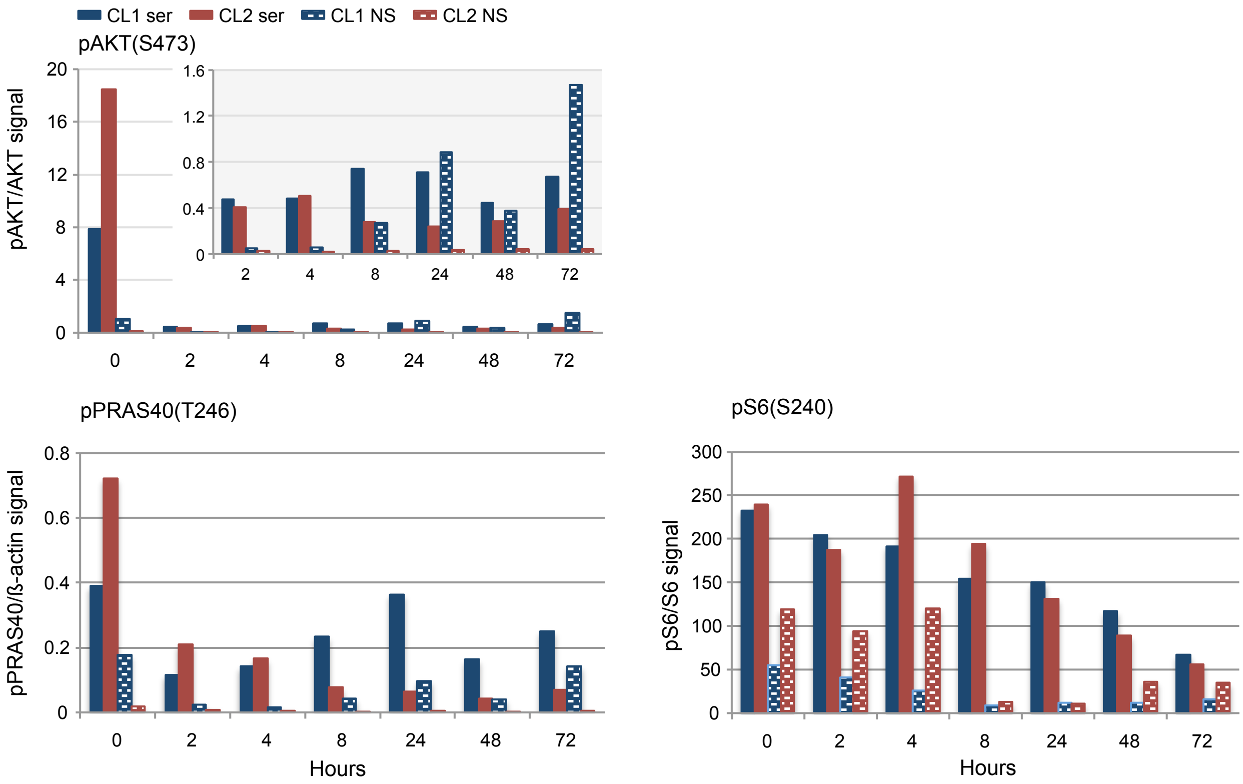

b

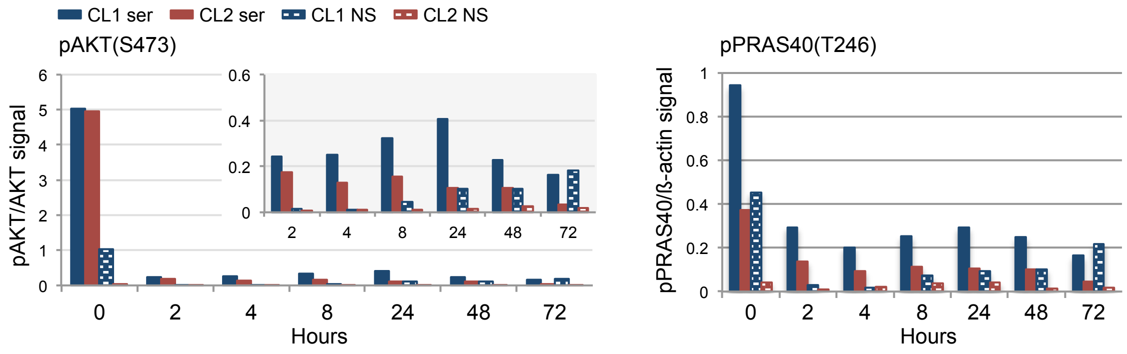

Supplementary Fig. 4. Additional experiments (a and b) showed pAKT and pPRAS40 levels were reduced within 2 hours of treatment with 125 nM ARQ 092. Both experiments were performed as described for Fig. 3. Insets in the pAKT histograms show the ratios from the ARQ 092-treated cells using a smaller scale. For both experiments, pAKT levels from CL1 grown without serum begin to rise at 8 hours compared to levels at the 2 or 4 hour time points. CL1, clone 1 (mutation-positive, blue bars); CL2, clone 2 (mutation-negative, red bars), ser, grown with serum (solid bars); NS, grown without serum (shaded bars).

Supplementary Figure 5

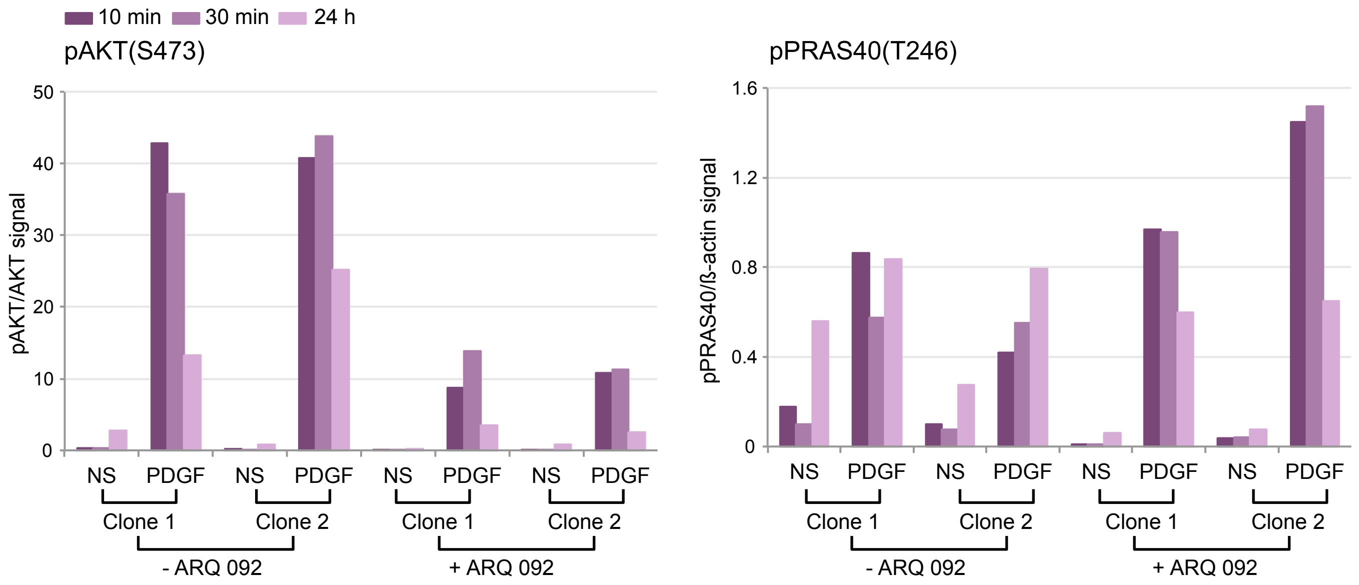

Supplementary Fig. 5. An additional experiment showed that platelet-derived growth factor-BB (PDGF-BB) stimulation in SCC treated with ARQ 092 resulted in elevated levels of pAKT and pPRAS40. This experiment was performed as described for Fig. 5. Cells were collected at 10 minutes, 30 minutes and 24 hours after PDGF-BB addition. Histograms depict ratios of indicated antibody pairs. pAKT levels increased 3- to >100-fold in ARQ 092-treated SCC and 5- to >100 fold in untreated SCC upon addition of PDGF-BB. pPRAS40 levels increased 9- to >85 fold in ARQ 092-treated SCC and two- to sevenfold in untreated SCC with PDGF-BB addition. NS, grown without serum; PDGF, PDGF-BB stimulated

Supplementary Figure 6

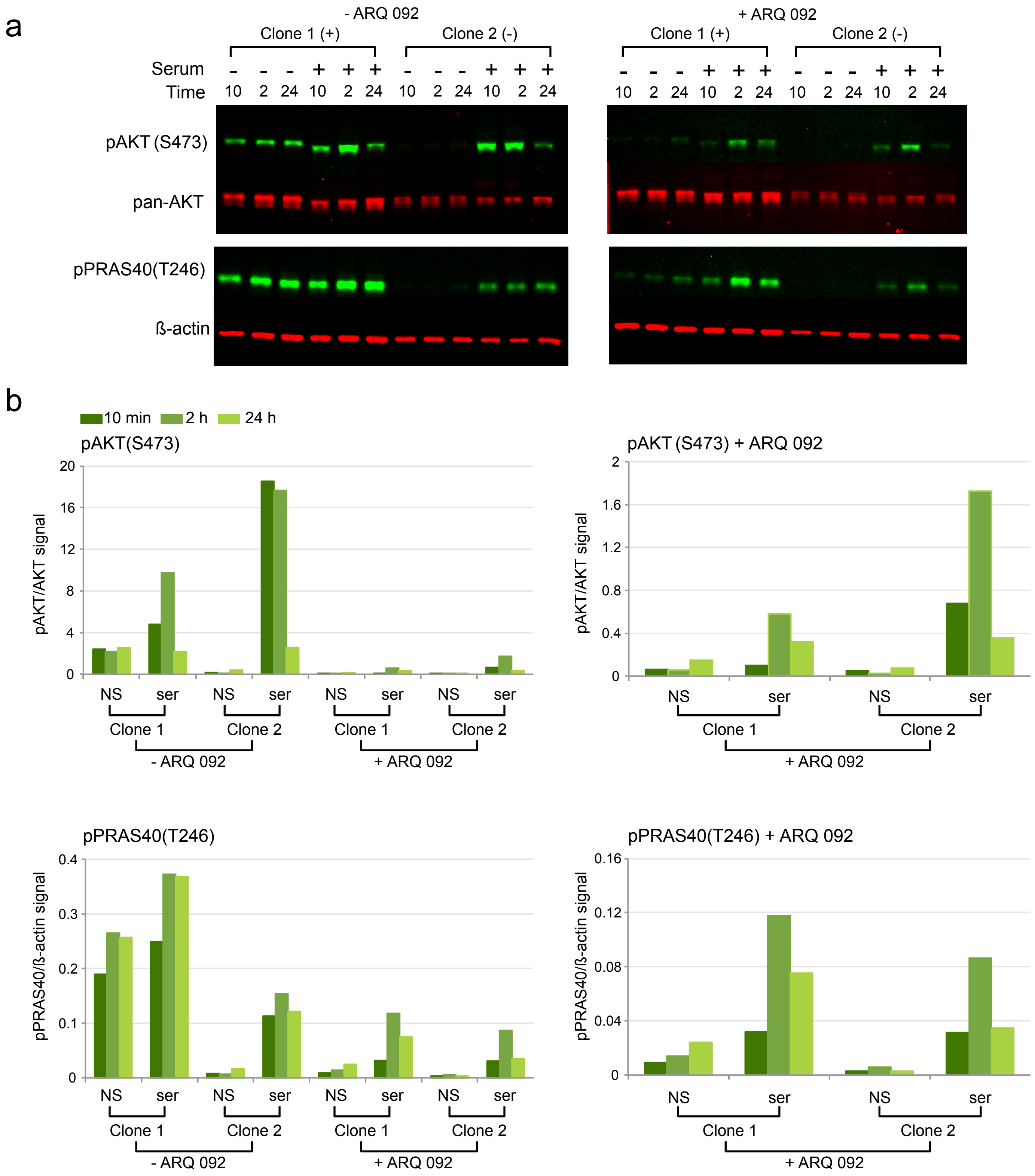

c

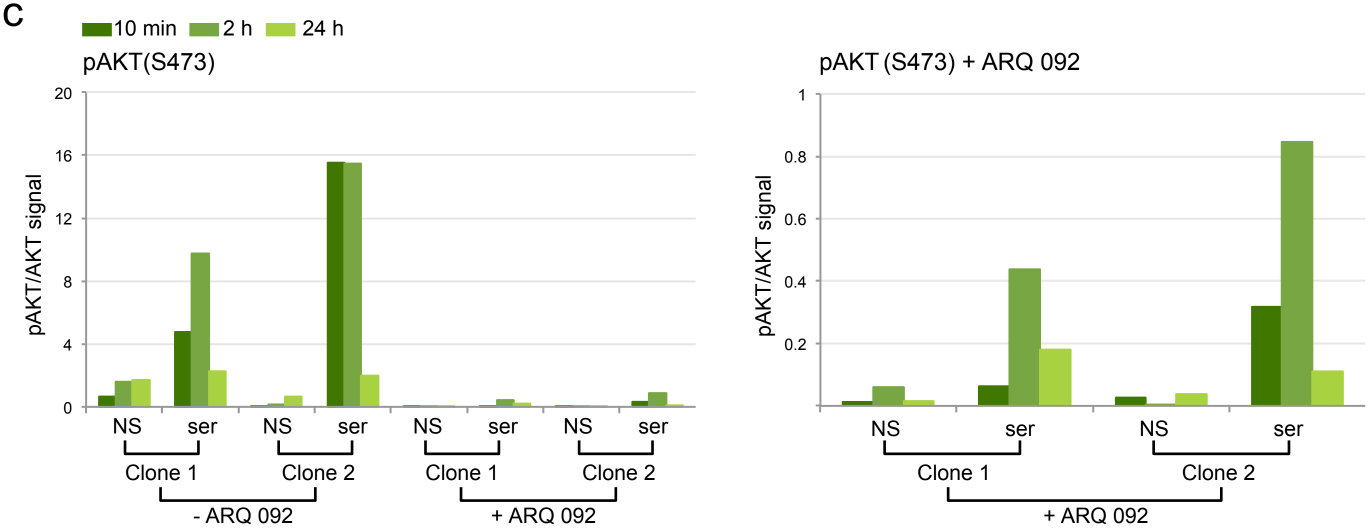

d

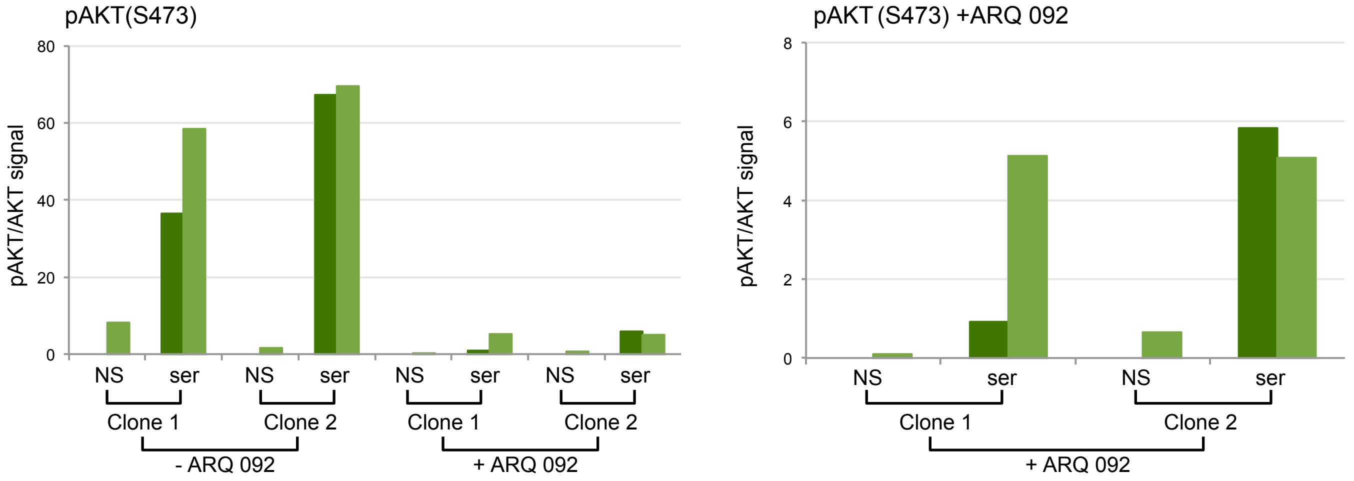

Supplementary Fig. 6. Serum stimulation in cells treated with ARQ 092 resulted in elevated levels of pAKT and pPRAS40. Mutation-positive (clone 1) and negative (clone 2) SCC were serum starved, treated +/- ARQ 092 and stimulated with 10% serum as described in the Methods. Cells were collected at 10 minutes, 2 hours and 24 hours after PDGF-BB addition. a) Infrared images of western hybridizations using the indicated antibodies. 10, 10 minutes, 2, 2 hours, 24, 24 hours. b) Histograms of the ratios of the infrared signals for each antibody pair. Signals for both clones +/- ARQ 092 treatment are graphed together on the left. Signals for ARQ 092-treated clones are also graphed on the right using a smaller scale. Serum addition increased pAKT levels 5- to >70-fold in ARQ 092-treated and 7- to >100-fold in untreated clone 2 cells compared to un-stimulated cells. Serum addition increased pAKT levels <2- to 10-fold in ARQ 092-treated and less than two- to fourfold in untreated clone 1 cells compared to un-stimulated cells. For pPRAS40, serum addition increased ARQ 092-treated clone 1 levels three- to eightfold and untreated clone 1 levels less than twofold. Serum addition increased pPRAS40 levels 11- to 15-fold in ARQ 092-treated and 8- to 25-fold in untreated clone 2 compared to un-stimulated cells. c and d) Histograms of pAKT/AKT ratios from additional serum stimulation experiments. Signals for ARQ 092-treated clones are graphed with untreated clones on left and separately on right using a smaller scale. For the experiment shown in d, un-stimulated cells were only collected at the 2-hour time point. Both experiments showed similar increases in pAKT levels upon serum stimulation in ARQ 092-treated SCC as was seen in the experiment shown in a and b. NS, grown without serum; ser, serum stimulated

Supplementary Figure 7

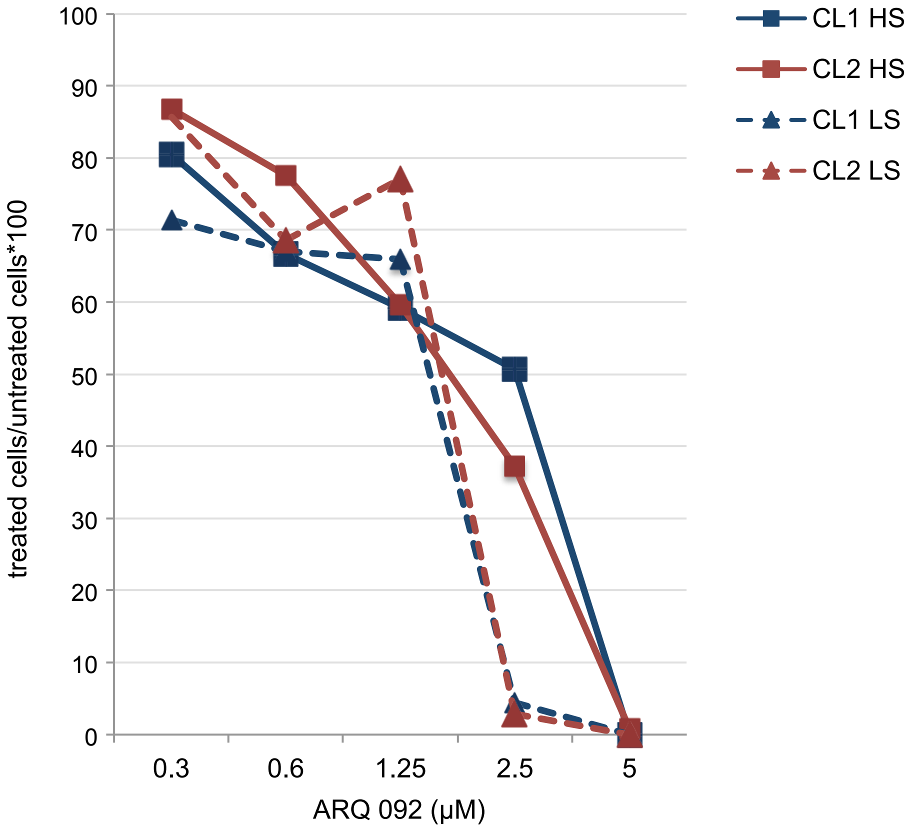

Supplementary Fig 7. Counting of cells showed that high concentrations of ARQ 092 were needed to reduce viability of cells from patients with Proteus syndrome. Mutation-positive and negative SCC were grown in high (10%) or low (0.5%) serum with increasing concentrations of ARQ 092 followed by manual cell counting as described in the Methods. CL1, clone 1 (mutation-positive); CL2, clone 2 (mutation-negative); HS, high serum; LS, low serum

Supplementary Figure 8

a

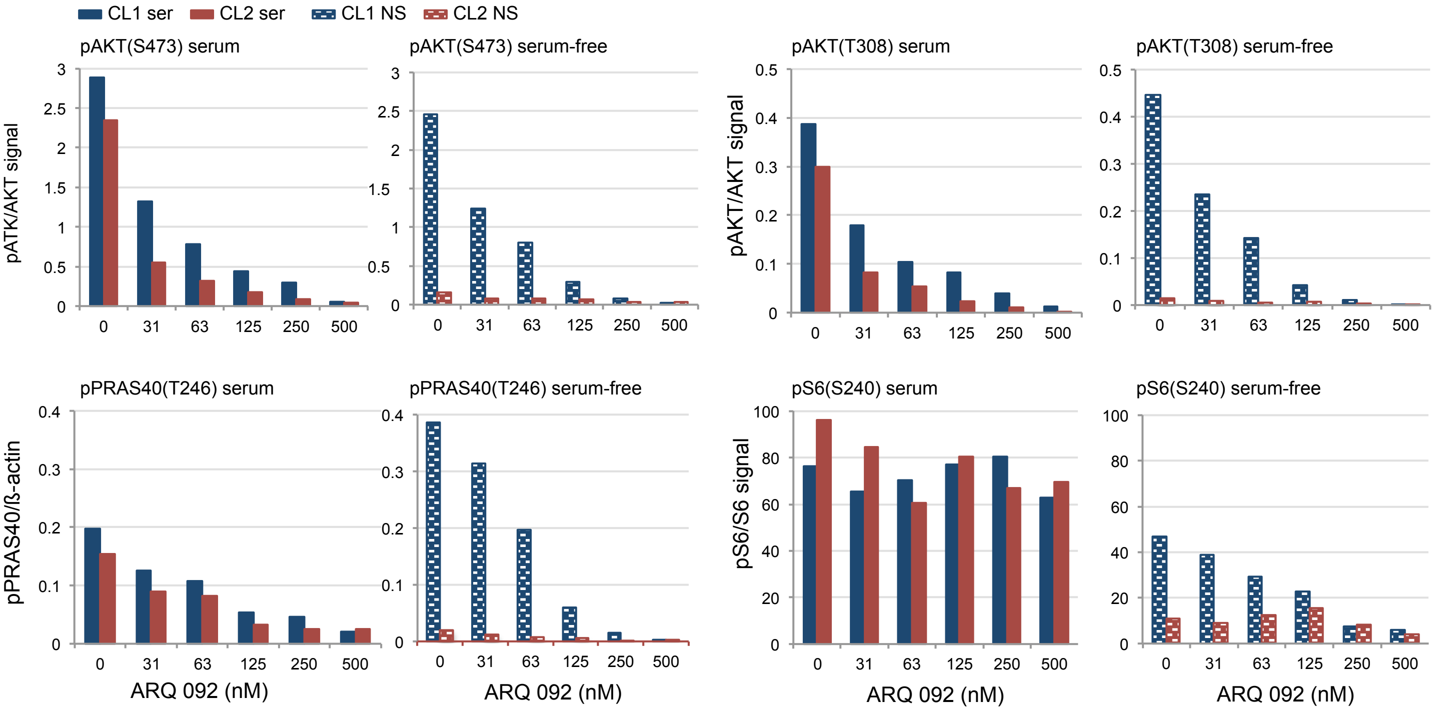

b

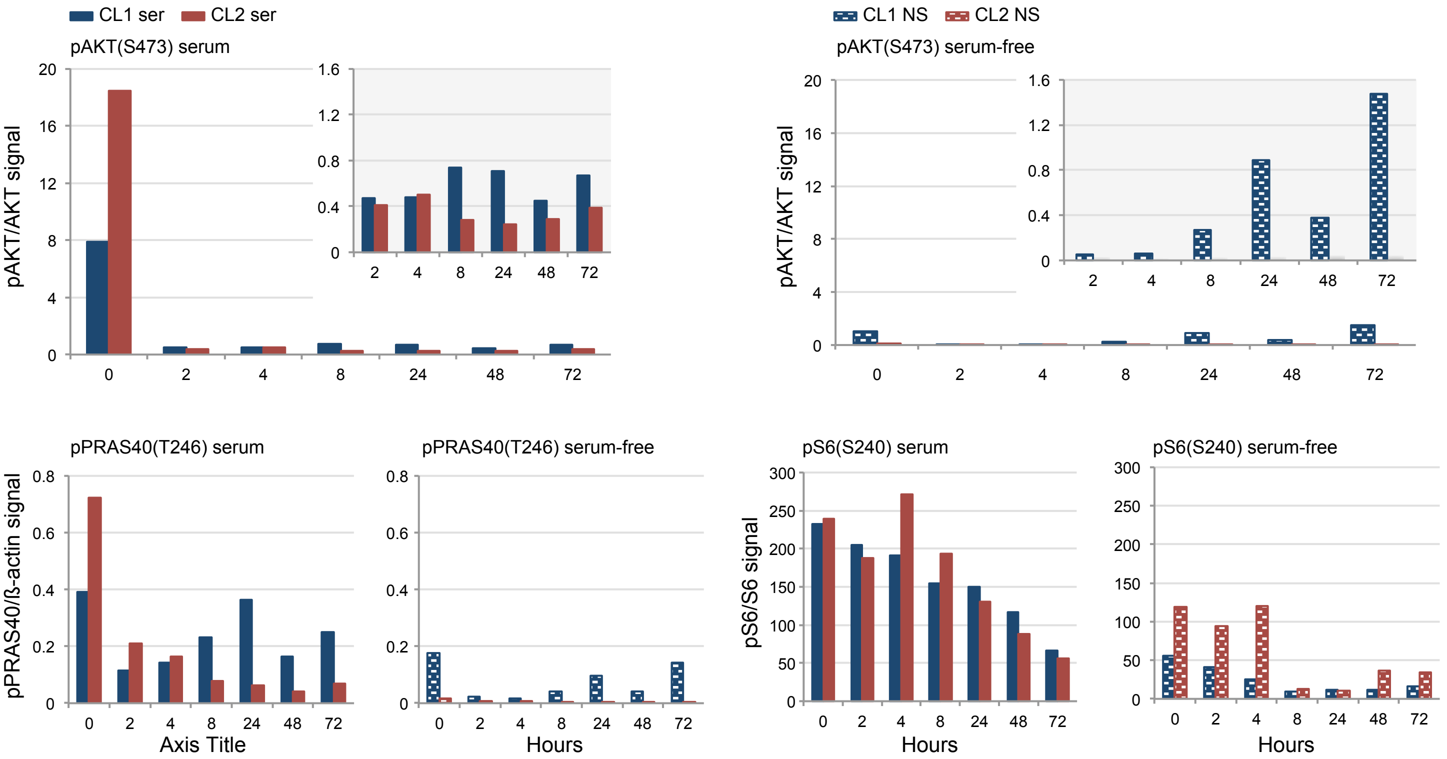

c

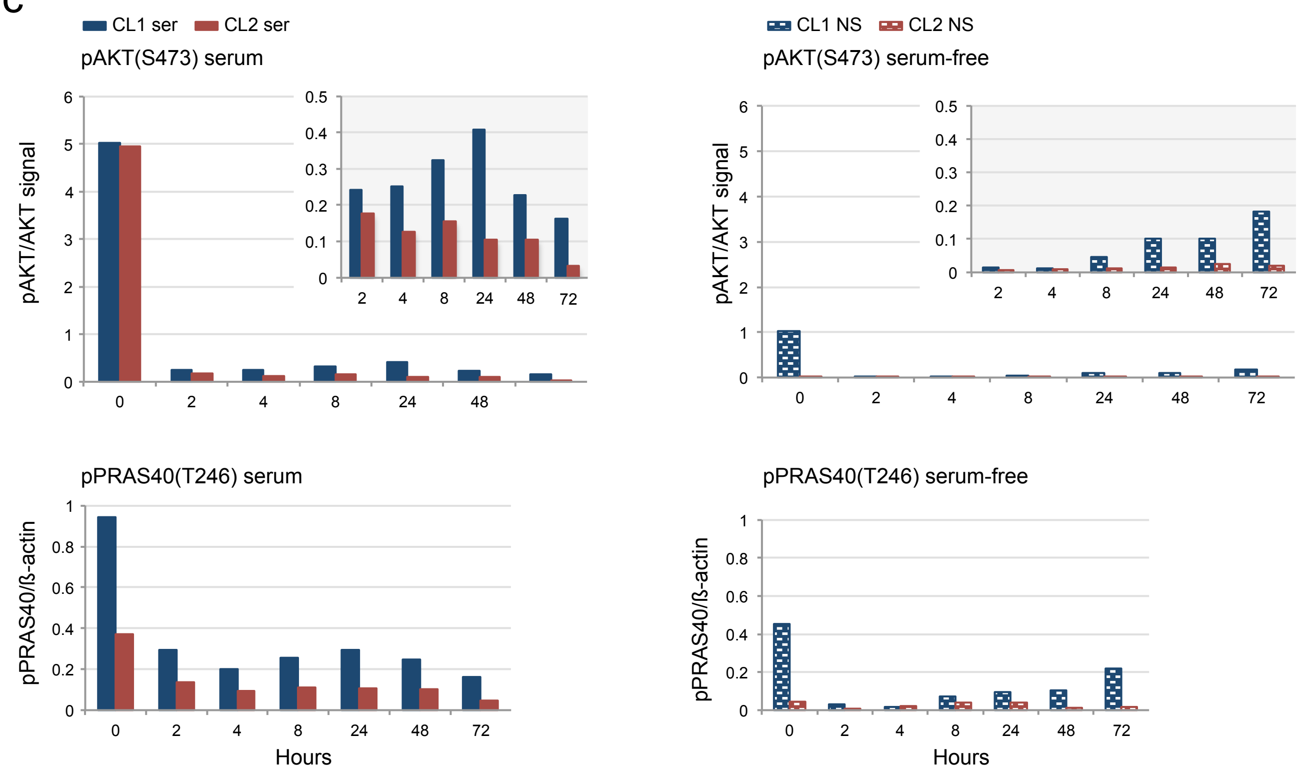

d

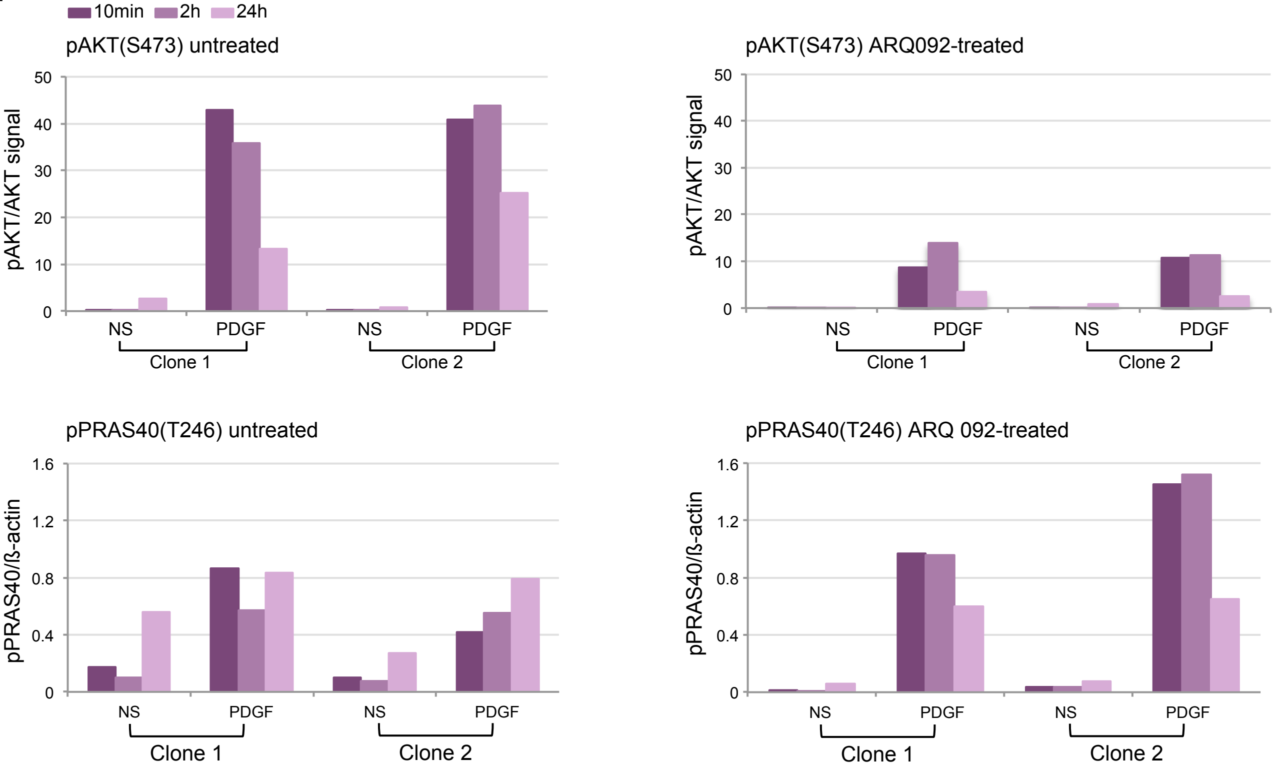

Supplementary Fig 8. Histograms of ratios of the infrared signals of the antibody pairs graphed by membrane. Ratios of signals were graphed by individual membrane for experiments when two membranes were used to assay the lysates. a) Data from Supplementary Fig. 1c. b) Data from Supplementary Fig. 4a. c) Data from Supplementary Fig. 4b. d) Data from Supplementary Fig. 5.
